# Supplementary material for: Pseudo‐repeats in doublecortin make distinct mechanistic contributions to microtubule regulation
Source: EMBO Rep. 2020 Oct 14;21(12):e51534. doi: 10.15252/embr.202051534 (PMC7726794; doi:10.15252/embr.202051534)
Supplement: Supplementary file 1 — Appendix [file EMBR-21-e51534-s001.pdf]

Appendix

Pseudo-repeats in doublecortin make distinct mechanistic contributions to microtubule regulation

Contents:

Appendix Figure S1
Appendix Figure S2
Appendix Figure S3
Appendix Figure S4
Appendix Figure S5
Appendix Figure S6
Appendix Table S1

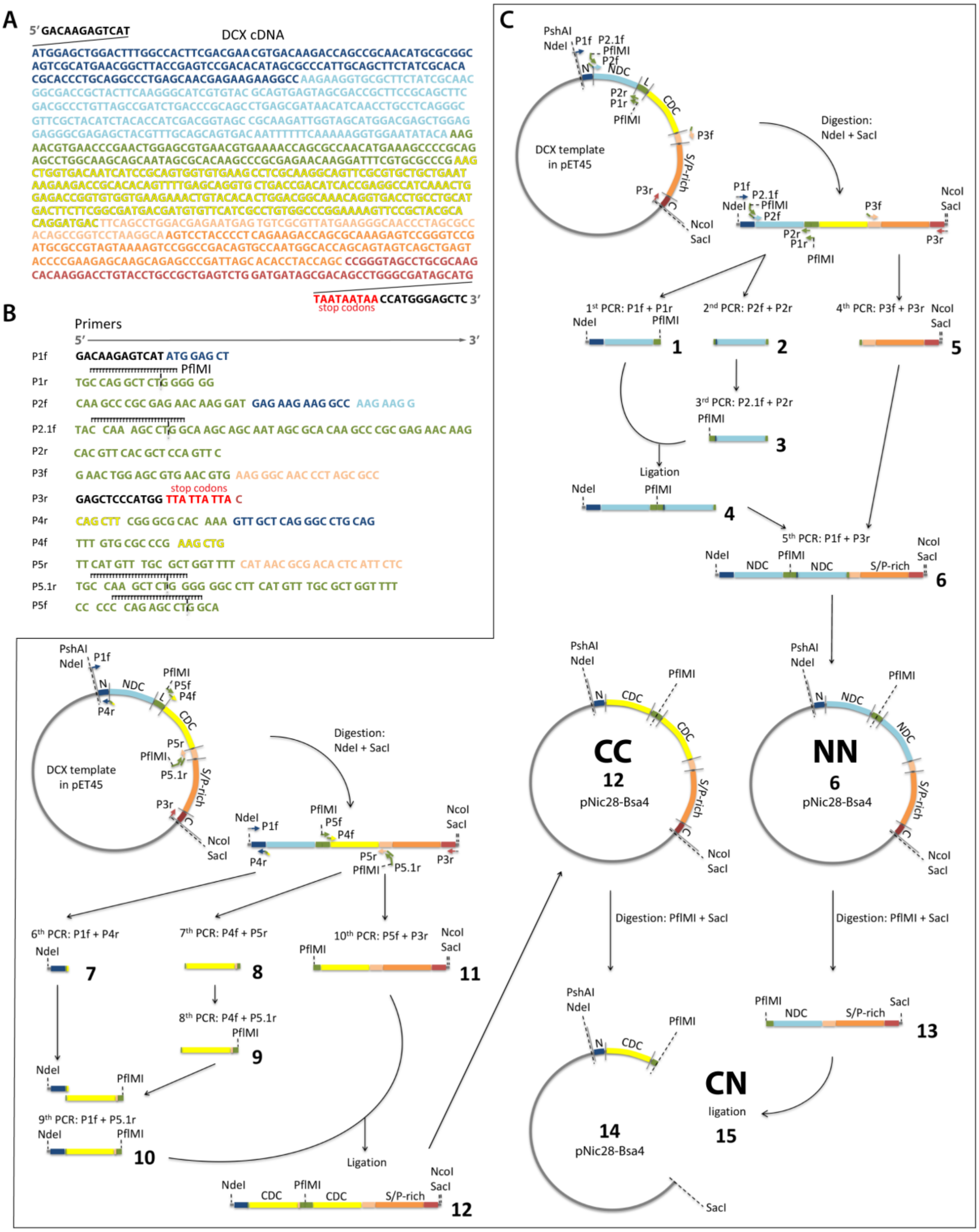

Appendix Figure S1. Cloning of the DCX constructs with repeated or swapped DC domains

A. Sequence of DCX isoform 2 cDNA used in this study with flanking sequences covering primer binding sites. Coloured according to region: navy blue, N-terminal region; blue, NDC; green, linker; yellow, CDC; light orange, post CDC region; orange, S/P-rich domain; dark red, C-terminal region.

B. List of primers used in the generation of the chimeras. Recognition and cleavage site for PflMI endonuclease is indicated.

C. Schematic representation of the PCR, restriction enzyme digestion and ligation steps (1-15) in the generation of the chimeras. NDC-NDC chimera, NN; CDC-CDC chimera, CC; CDC-NDC chimera, CN.

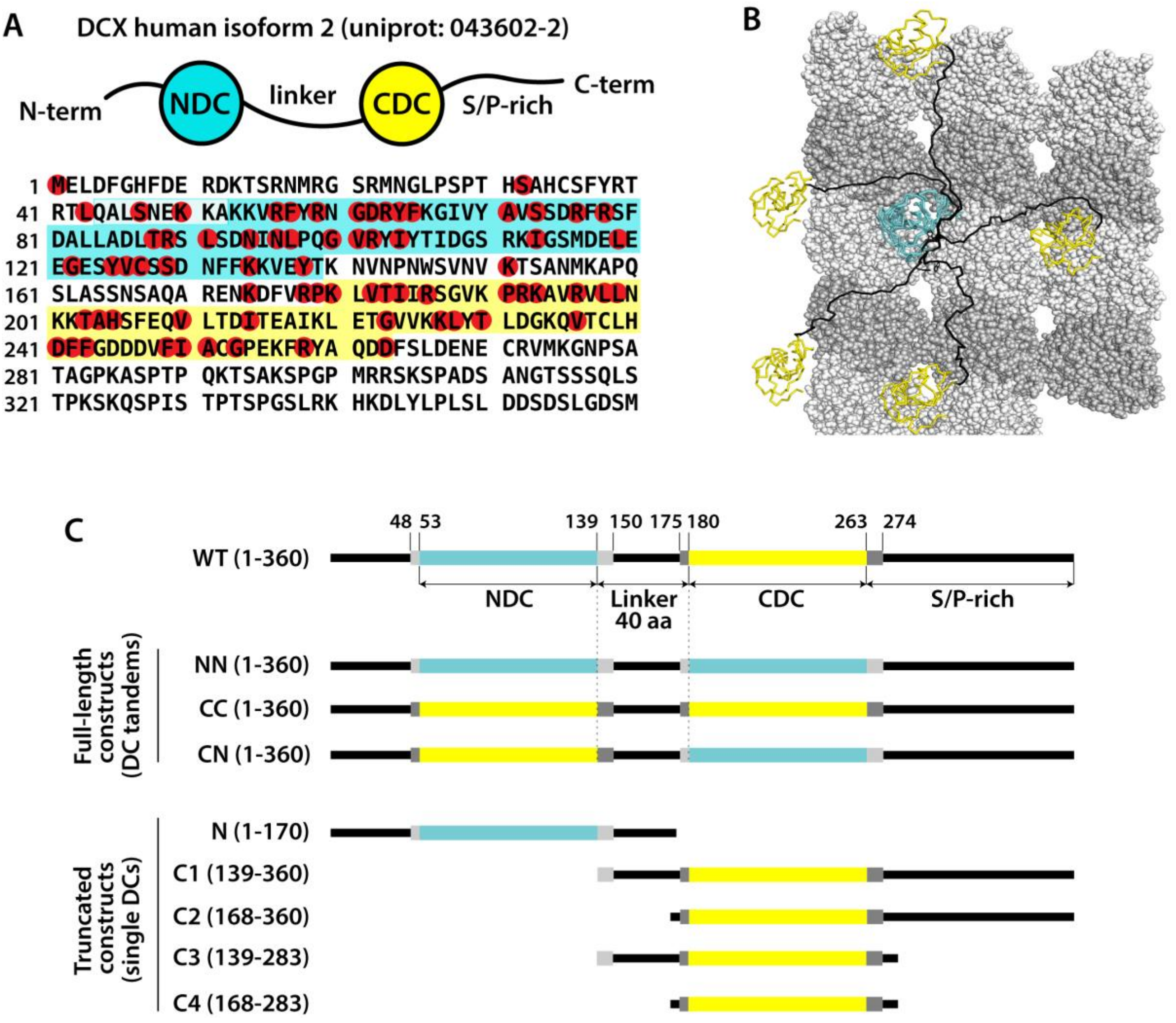

Appendix Figure S2. Characteristics of the DCX structure and details about the generated constructs

A. Schematic of DCX structural regions and the location of symptomatic missense mutation sites according to the OMIM.org database**.**

B. Manual modelling (*sculpting*) in PyMOL (Schrödinger) shows that the length of the DCX linker region (black) is in principle compatible with multiple modes of DCX binding to MT lattice. NDC, blue; CDC, yellow; α-tubulin, dark grey; β-tubulin, light grey.

C. List of DCX constructs generated in this study with database (Uniprot.org) DC domain boundaries indicated with colour and the cloning boundaries (including flanking regions) indicated with grey zones. We took care to maintain the length of the linker (dotted lines) in all full-length constructs (DC tandems).

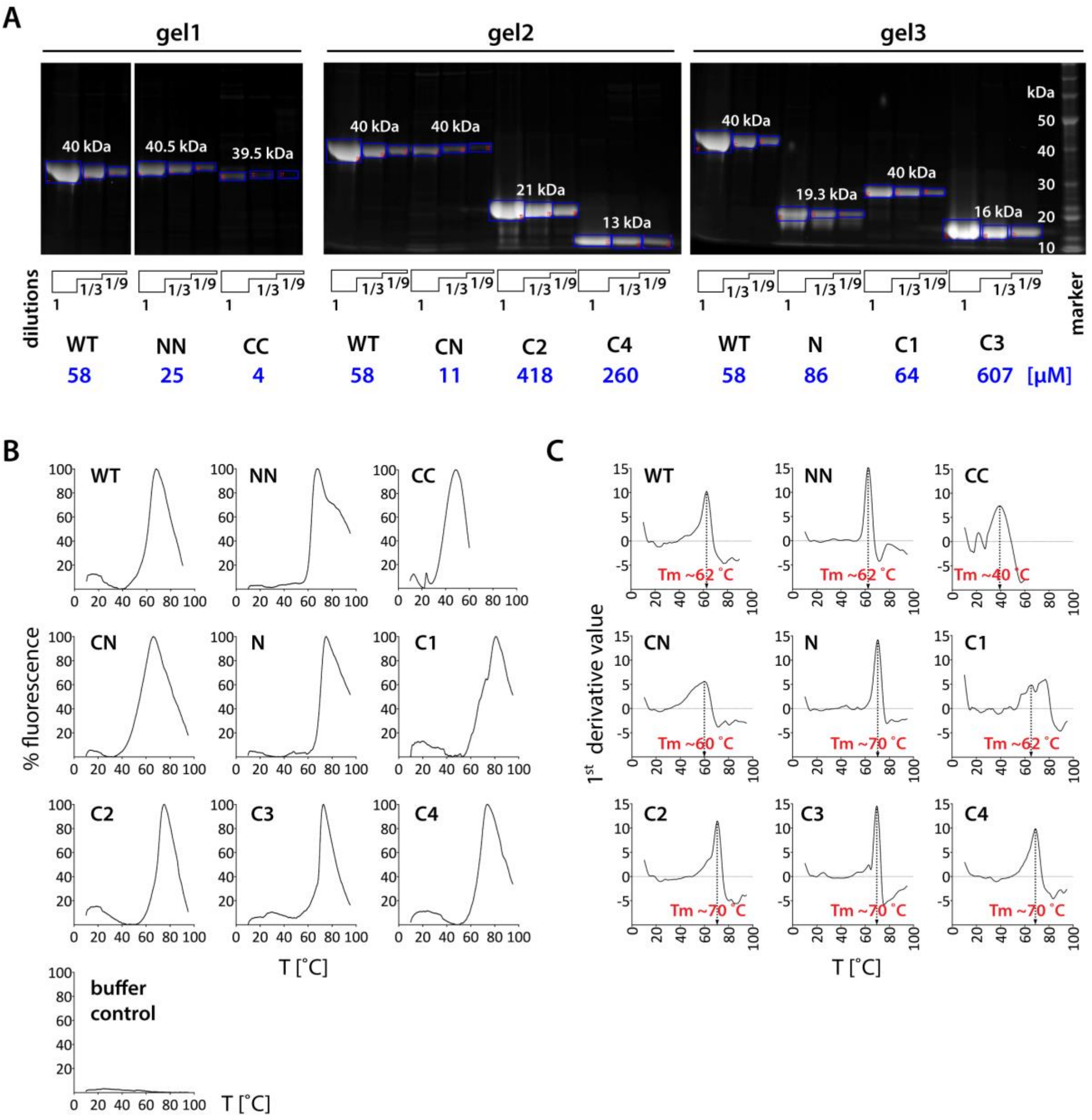

Appendix Figure S3. Purity and thermal stability of the generated constructs

A. Purity of DCX proteins was assessed by SDS-PAGE under reducing conditions using Bolt Bis-Tris Plus gels (Thermo Fisher Scientific). Peak fractions from the S200 gel filtration column (final purification step) were pooled, concentrated using Vivaspin columns (GE Healthcare) and run neat and at two indicated dilutions (1, 1/3 and 1/9). All gels included WT sample as an internal standard. Some lanes in gel 1 were removed as irrelevant to this study. The gels were stained with SYPRO Orange Protein Gel Stain (Sigma-Aldrich) and scanned using Fujifilm FLA-3000 Fluorescence Laser Imaging Scanner. The intensities of the bands were quantified using FiJi (https://fiji.sc/ (Schindelin et al., 2012); as indicated with the blue boxes with red annotations), multiplied according to their dilution factor and averaged together. Protein concentrations are derived from the band intensity ratio to the WT internal standard, which was measured by absorbance at 280 nm wavelength.

B. Normalized thermal shift (ThermoFluor) plots. The inflection point on the rising curve is the melting temperature (T_m).

C. First derivative plots to identify T_m with the peaks, corresponding to the inflection points in the thermal shift curves in (B).

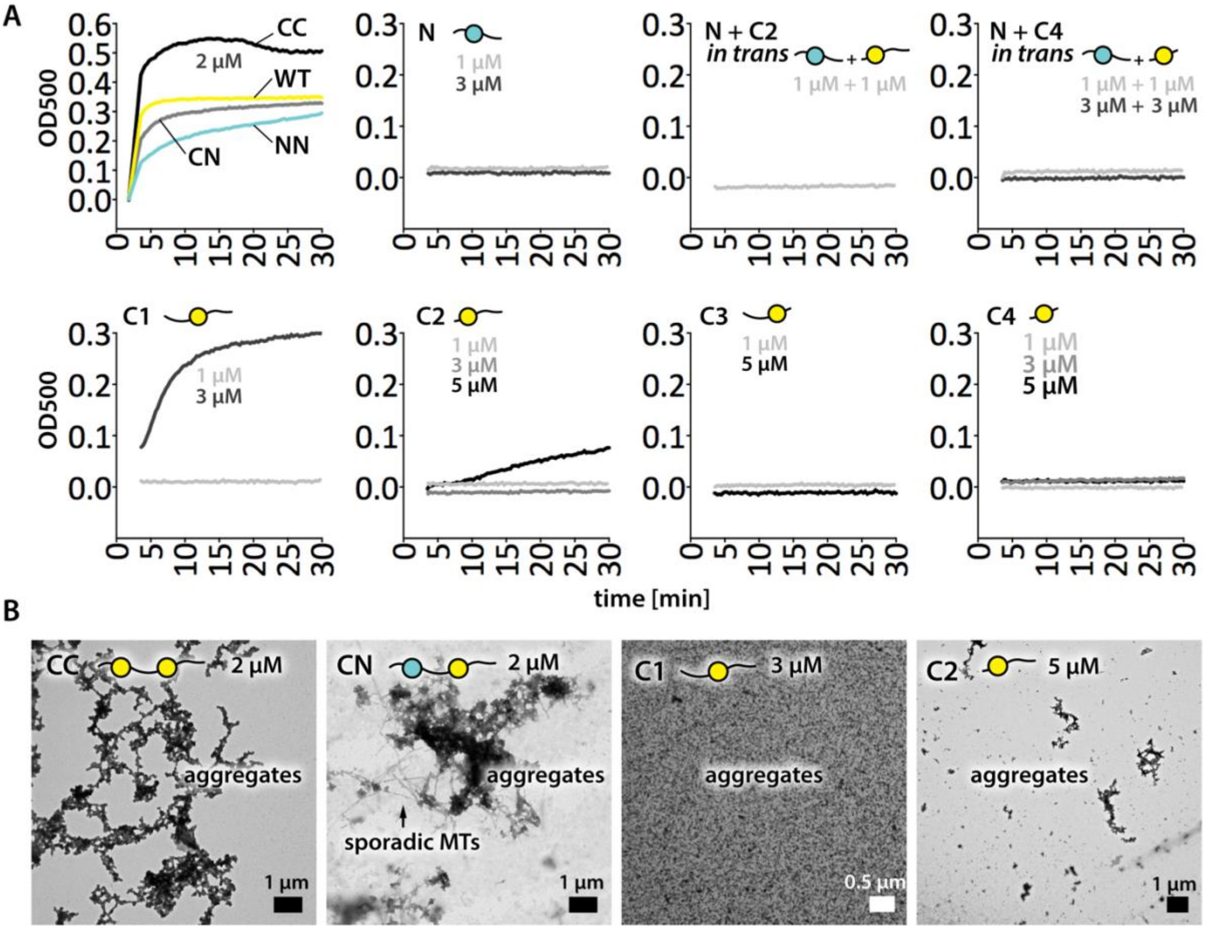

Appendix Figure S4. MT nucleation, abilities of different DCX constructs

A. Turbidity assay plots. 5 μM tubulin was mixed with indicated concentrations of various DCX constructs and light scattering was measured at 500 nm wavelength (OD500).

B. Direct verification of turbidity assay results by negative stain EM imaging of selected samples from (A). Protein aggregation increases sample turbidity and can be confused for MT nucleation in this assay.

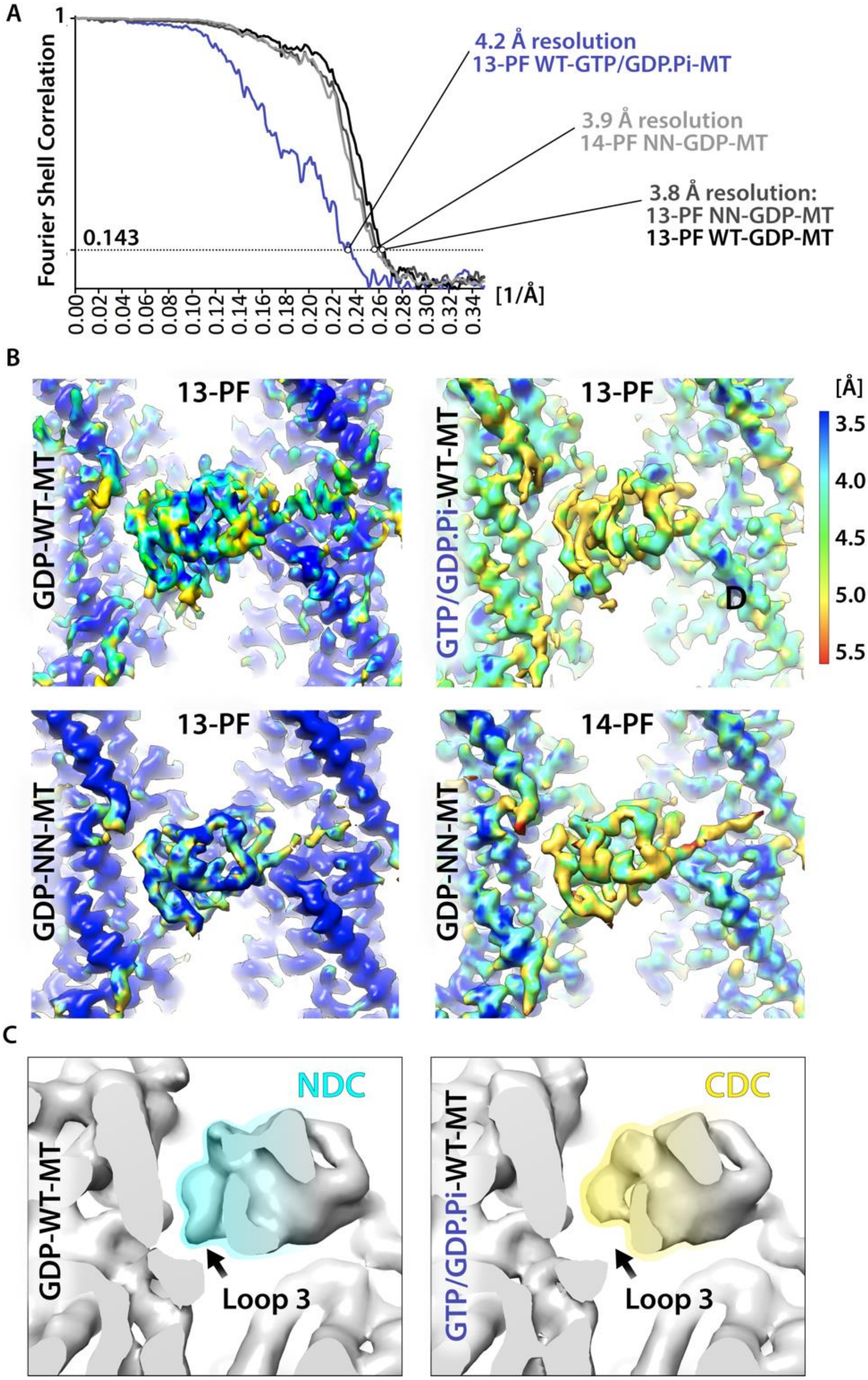

Appendix Figure S5. Global and local resolution estimation and low-pass filtering of cryo-EM reconstructions
A. Fourier Shell Correlation (FSC) plots and resolution estimation according to the 0.143 cut-off criterion.

B. Local resolution maps obtained with ResMap (Kucukelbir et al., 2014).
C. Fourier filtering of both GDP-WT-MT and GTP/GDP.Pi-WT-MT maps to 8 Å resolution for comparisons of their overall folds (backbones and secondary structures) visually discriminates the DC domain density from each reconstruction (distinct colour shading), based on loop 3, the major differentiating feature. View orientation as in Figure 2.

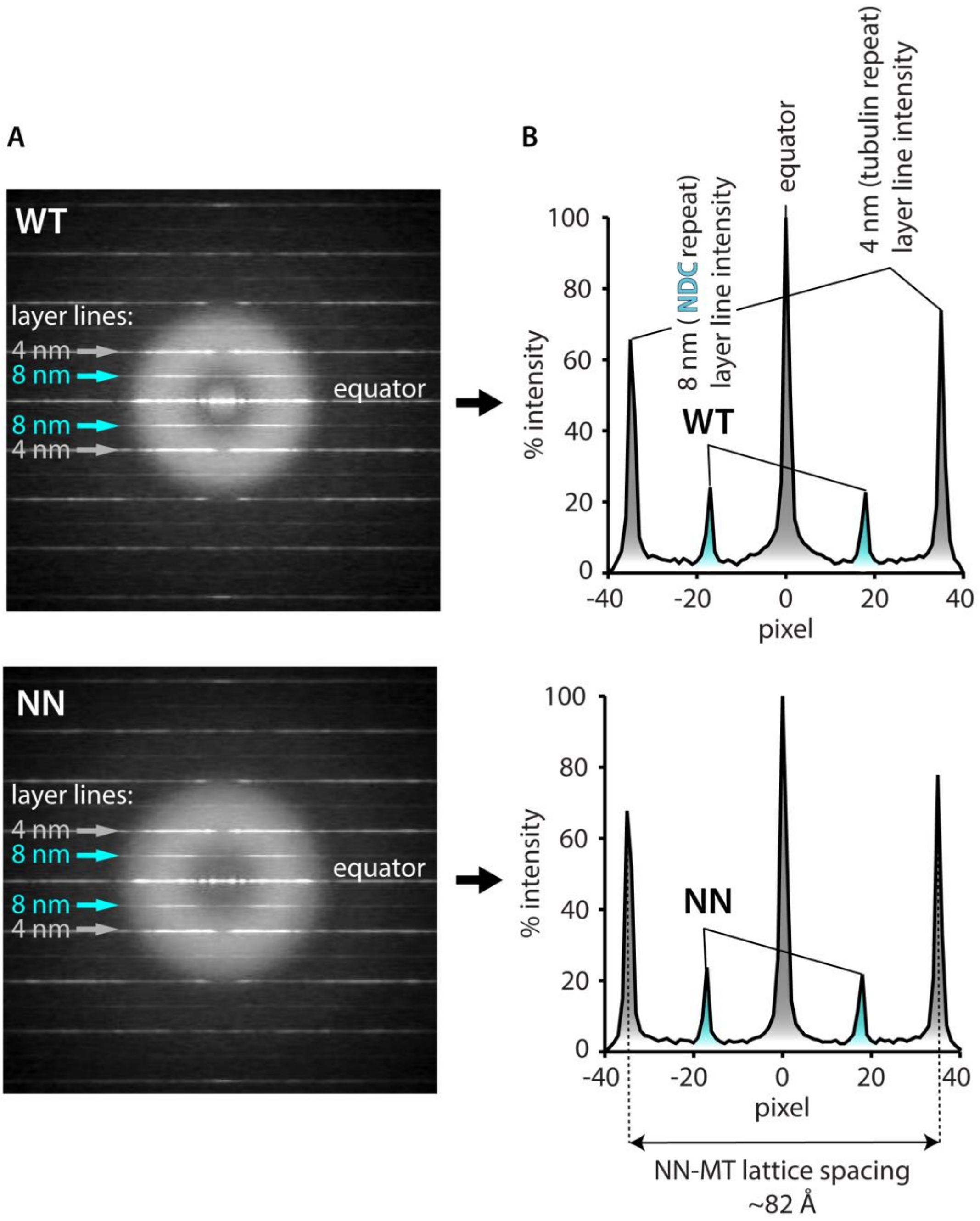

Appendix Figure S6. Fourier transformation of MT lattices decorated with WT or NN
A. Averaged Fourier transforms of WT- and NN-decorated GDP-MT segments. Fourier transform of a DCX-MT image shows layer lines corresponding to 4 nm periodicity (tubulin subunits) and 8 nm periodicity (DC domain binding at MT lattice vertices).
B. Layer line intensities plotted using FiJi (fiji.sc). The plots are normalized against the equator intensity expressed as 100%. Relative MT lattice decoration by WT and NN can be assessed by the DC domain:tubulin layer line ratio. Similarly to WT-MT lattice, NN-MT lattice is compacted (~82 Å spacing = tubulin repeat distance), as expected for the GDP state (Manka and Moores, 2018).

Appendix Table S1. Symptomatic missense mutations of doublecortin reported in humans

Mutation	Clinical significance	Condition	Review status	Last reviewed
Pro64Leu*	LP	Lis	s	Jan 1 2018
Met1Thr	P	H	s	Feb 8 2013
Ser32Asn	P	H	s	Feb 8 2013
Leu43Ser	P	H	s	Feb 8 2013
Ser47Arg	P	Lis|SLH	n.a.	Jan 9 1998
Lys50Asn	P	H	s	Feb 8 2013
Arg56Pro	LP	H	s	Apr 2 2014
Phe57Cys	LP	H	s	Feb 8 2013
Arg59Leu	P	Lis|SLH	n.a.	Jan 9 1998
Arg59His	P	H	s	Feb 8 2013
Gly61Glu	P	H	s	Feb 8 2013
Asp62Asn	P	Lis|SLH	n.a.	Jan 9 1998
Asp62Gly	LP	H	s	Feb 8 2013
Arg63Cys	LP	H	s	Feb 8 2013
Tyr64Cys	LP	H	s	Feb 8 2013
Tyr145Asn*	LP	Lis	s	Sep 1 2017
Phe65Leu	LP	H	s	Feb 8 2013
Ala71Ser	P	Lis|SLH	n.a.	Jan 28 2003
Ser73Phe	P	H	s	Feb 8 2013
Arg76Cys	LP	H	s	Apr 16 2013
Arg78His	P	SLH	n.a.	Sep 1 1999
Arg78Leu	P	H	s	Feb 8 2013
Arg78Cys	P	H	s	Feb 8 2013
Arg89Pro	P	H	s	Feb 8 2013
Arg89Gly	P	SLH	n.a.	Sep 1 1999
Leu91Pro	P	H	s	Feb 8 2013
Asn94Asp	P	a.c.g.	s	Feb 8 2013
Asn96Lys	P	F	s	Dec 3 2017
Leu97Arg	P	H	s	Feb 8 2013
Gly100Glu	LP	H	s	Feb 8 2013
Gly100Val	P	H	s	Feb 8 2013
Val101Met	P	H	s	Feb 8 2013
Arg102Cys	P	H	s	Feb 8 2013
Arg102His	P|LP	H|n.p.	m n.c.	Sep 30 2016
Arg102Gly	P	n.p.	s	Jan 22 2016
Ile104Phe	P	H	s	Feb 8 2013
Ile113Phe	LP	H	s	Feb 8 2013
Gly122Arg	P	H	s	Feb 8 2013

Tyr125His	P	Lis|SLH	n.a.	Jan 9 1998
Val126Asp	LP	H	s	Feb 8 2013
Cys127Tyr	LP	H	s	Feb 8 2013
Ser129Leu	P	H	s	Aug 7 2013
Tyr138His	P	H	s	Feb 8 2013
Tyr138Cys	P	H	s	Feb 8 2013
Lys151Glu	P	H	s	Feb 8 2013
Lys174Glu	P	H	s	Feb 8 2013
Arg178Cys	P	H	s	Feb 8 2013
Arg178Gly	P	H	s	Feb 8 2013
Arg178Leu	P	H	s	Feb 8 2013
Lys180Glu	P	H	s	Feb 8 2013
Val182Phe	P	Lis	s	Aug 23 2016
Ile184Asn	LP	H	s	Feb 8 2013
Arg186His	c.i.	Lis|H	c.i.	Sep 1 2017
Arg186Cys	P	H|n.p.	m n.c.	Mar 30 2017
Arg186Leu	P	H	s	Feb 8 2013
Pro191Arg	P	H|n.p.	m n.c.	May 30 2017
Pro191Thr	LP	H	s	Feb 8 2013
Arg192Trp	P	Lis|SLH|H|n.p.	m n.c.	Dec 22 2016
Arg196Ser	P	H	s	Feb 8 2013
Arg196His	P	Lis|SLH |H	s	Feb 8 2013
Arg196Gly	P	H	s	Feb 8 2013
Arg196Cys	P|LP	H|n.p.	m n.c.	Jun 12 2017
Leu199Pro	LP	H|n.p.	m n.c.	Aug 8 2016
Thr203Arg	P	Lis|SLH	n.a.	Jan 9 1998
Thr203Ala	P	H	s	Feb 8 2013
Thr203Lys	P	H	s	Feb 8 2013
Ala204Asp	P	H	s	Feb 8 2013
His205Leu	LP	H	s	Feb 8 2013
Val210Phe	P	H	s	Feb 8 2013
Ile214Thr	P	H	s	Feb 8 2013
Gly223Arg	P|LP	H|n.p.	m n.c.	Oct 13 2016
Leu228Pro	P	H	s	Feb 8 2013
Thr230Pro	LP	H	s	Feb 8 2013
Phe242Ile	LP	H	s	Aug 27 2013
Phe249Cys	LP	H	s	Feb 8 2013
Ala251Ser	P	H	s	Feb 8 2013
Ala251Asp	LP	n.p.	s	Jul 28 2015
Gly253Arg	LP	n.p.	s	Jul 24 2017
Arg258Pro	LP	H	s	Feb 8 2013

Mutations in grey lie outside of DC domains; a.c.g., abnormal cortical gyration; c.i., conflicting interpretations; F, fucosidosis; H, heterotopia; Lis, lissencephaly; LP, likely pathogenic; m, multiple submitters; n.a., no assertion; n.c., no conflicts; n.p., not provided; P, pathogenic; s, single submitter; SLH, subcortical laminar heterotopia;

*transcript variant 1, with 81 amino acids longer N-terminal sequence (starting with Met -81 with respect to transcript variant 2 relevant in this study), Pro64 in transcript 1 does not exist in DCX isoform from this study and Tyr145 is equivalent with Tyr64 in DCX isoform from this study.

References

Kucukelbir, A., Sigworth, F.J., and Tagare, H.D. (2014). Quantifying the local resolution of cryo-EM density maps. Nat. Methods *11*, 63–65.

Manka, S.W., and Moores, C.A. (2018). Microtubule structure by cryo-EM: snapshots of dynamic instability. Essays Biochem. *62*, 737–751.

Schindelin, J., Arganda-Carreras, I., Frise, E., Kaynig, V., Longair, M., Pietzsch, T., Preibisch, S., Rueden, C., Saalfeld, S., Schmid, B., et al. (2012). Fiji: an open-source platform for biological-image analysis. Nat. Methods *9*, 676–682.
