# Supplementary material for: Pseudo‐repeats in doublecortin make distinct mechanistic contributions to microtubule regulation
Source: EMBO Rep. 2020 Oct 14;21(12):e51534. doi: 10.15252/embr.202051534 (PMC7726794; doi:10.15252/embr.202051534)
Supplement: Supplementary file 2 — Expanded View Figures PDF [file EMBR-21-e51534-s002.pdf]

Expanded View Figures

Figure EV1. MT populations stabilized by WT and NN and the influence of all DCX constructs on MT dynamic instability parameters.

A Example cryo-EM micrographs of WT-MTs and NN-MTs presenting moiré patterns corresponding to different (13-PF or 14-PF) MT architectures. 14-PF MTs are only found in the NN-MT sample.

B Example Fourier filtering in FiJi (https://fiji.sc/), depicting differences in moiré patterns of 13-PF and 14-PF MTs, used as a diagnostic in this study.

C Quantification of PF number distribution in WT-MT and NN-MT samples.

D–G Influence of different DCX constructs on MT dynamic instability parameters measured with TIRF microscopy. Tubulin concentration was 10 μM in all experiments.

Data Information: In (D–G) data are presented as box & whiskers plots; whiskers, the minimum and the maximum measured value; box, interquartile range (between the 25th and 75th percentile); central line, the median value (50th percentile). Indicated sample sizes: number of MTs and, where appropriate, events. Significant differences compared to the reference value of tubulin alone according to the multiplicity adjusted *P* values: ****$P < 0.0001$; **$P < 0.01$; ns, not significant ($P > 0.05$; one-way ANOVA verified by Holm–Sidak's multiple comparisons test).

 © 2020 The Authors

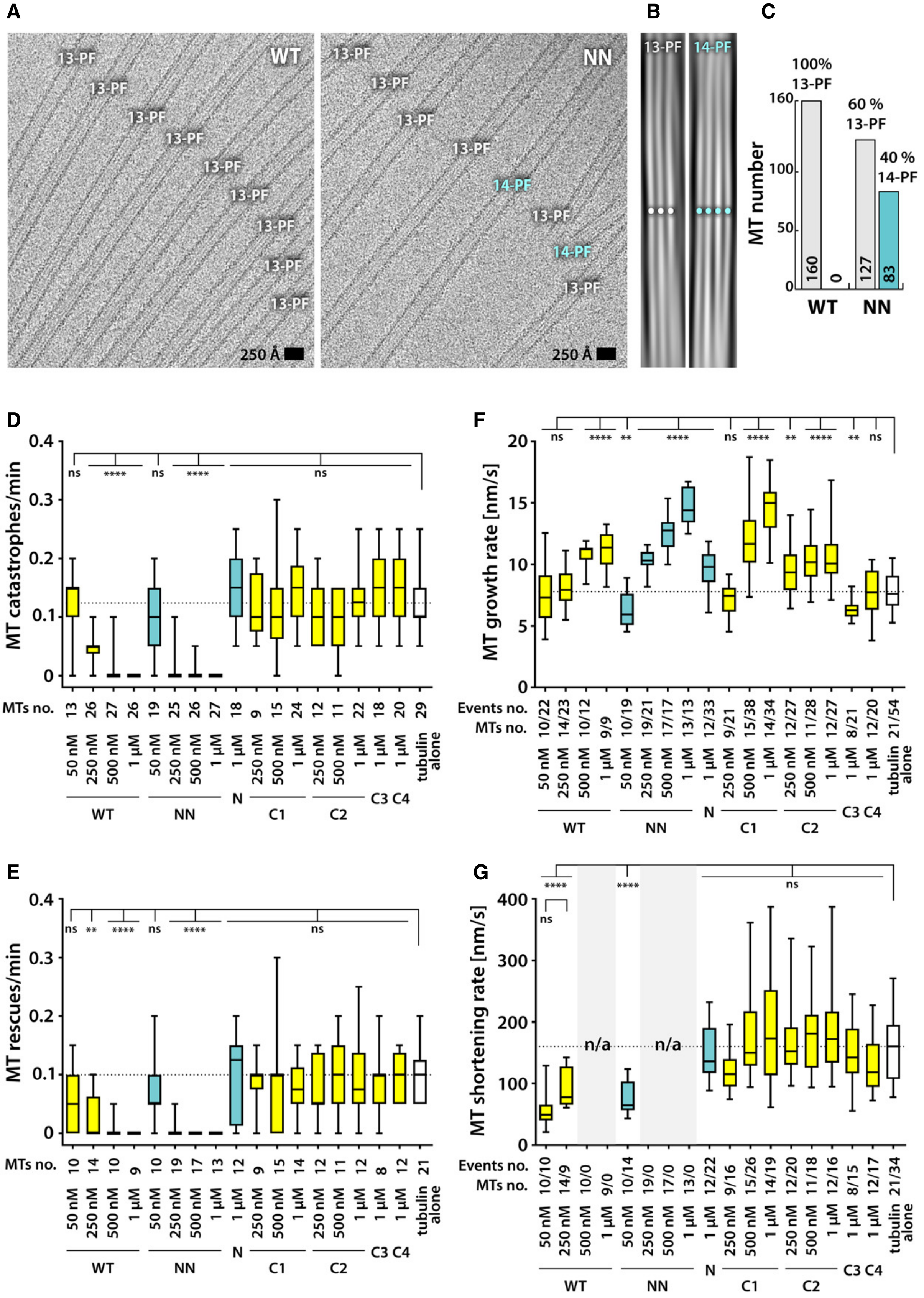
A
B
C
WT
NN
13-PF
14-PF
250 Å
100% 13-PF
60 % 13-PF
40 % 14-PF
MT number
160
0
127
83
D
MT catastrophes/min
E
MT rescues/min
F
MT growth rate [nm/s]
G
MT shortening rate [nm/s]
MTs no.
Events no.
n/a
ns
****
**
50 nM
250 nM
500 nM
1 µM
tubulin alone
N
C1
C2
C3 C4

Figure EV1.

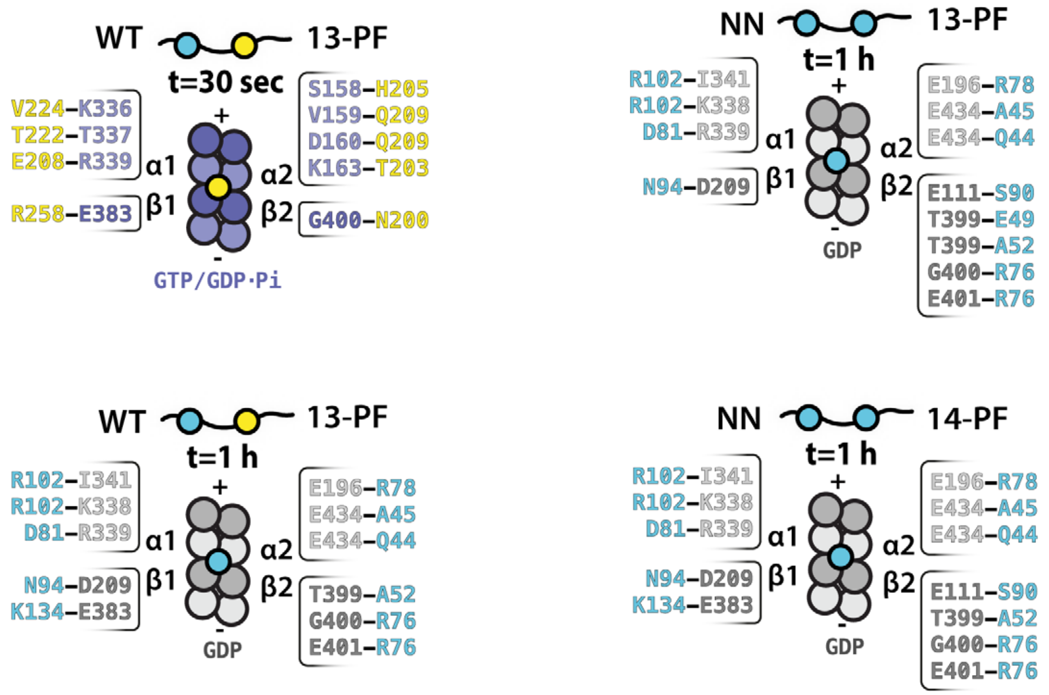

Figure EV2. Summary of the resolved WT-MT and NN-MT polar contacts.

Schematic diagrams of DC domains bound in the vertices of four tubulin dimers in WT-MTs and NN-MTs, according to our cryo-EM reconstructions, including listing of polar contacts (H-bonds and/or salt bridges) made by these DC domains with indicated tubulin subunits. The contacting residues were identified with help of PDBePISA (http://www.ebi.ac.uk/msd-srv/prot_int/cgi-bin/piserver). NDC, blue; CDC, yellow.

© 2020 The Authors

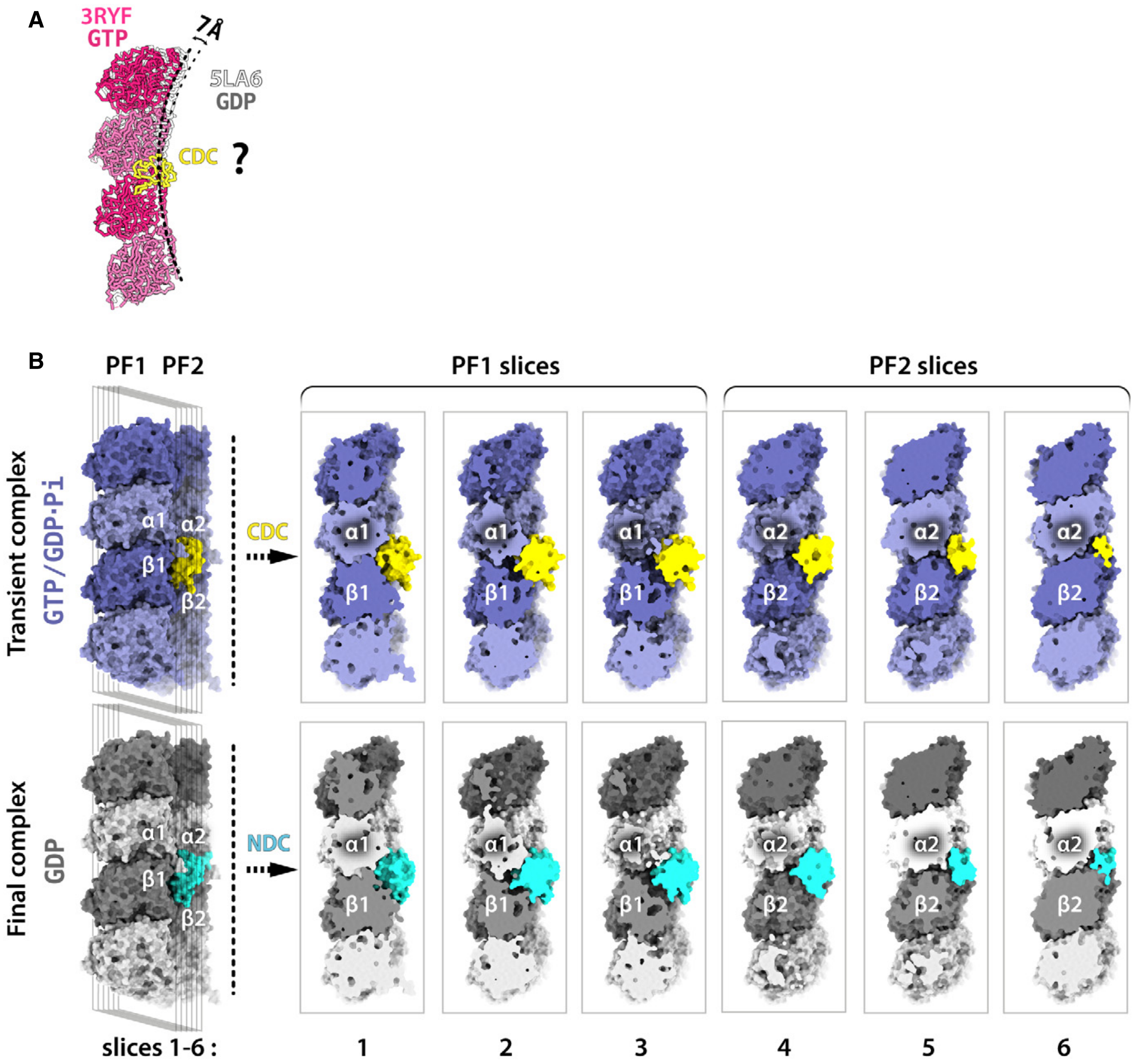

Figure EV3. Comparisons of putative CDC-tubulin, CDC-MT, and NDC-MT complexes.

A Alignment of X-ray structures of longitudinally associated bent tubulin dimers: 3RYF (GTP state; pink) and 5LA6 (GDP state; gray) shows different degrees of curvature between complexes of tubulin in different nucleotide states. It is hypothesized (question mark) that CDC binding at the junction of tubulin assembly may further limit tubulin bending.

B Series of cross-sections through DC domain complexes with MT at different stages of MT assembly.

Figure EV4. Tubulin polymerization products in the presence of WT and NN.

A Example cryo-EM micrographs showing WT-MTs and NN-MTs surrounded by other tubulin polymerization products present at indicated polymerization time points. Arrows indicate incomplete MTs.

B Close-up views of single and laterally associated non-MT curved tubulin protofilaments (PFs) showing various numbers and degrees of curvature, indicated with offset pink arches.

Data information: Scale bar, 200 Å.

© 2020 The Authors	

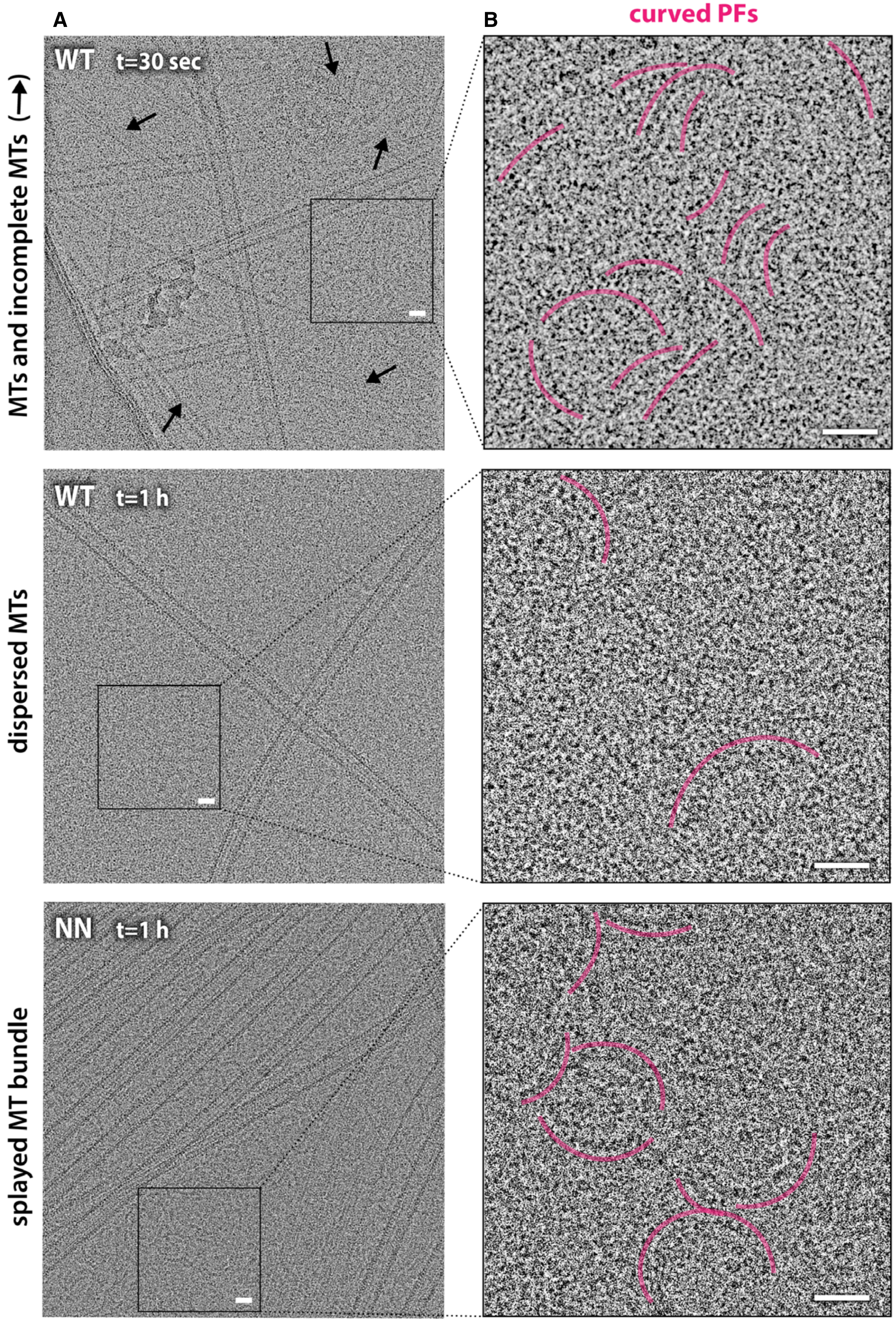

Figure EV4.

© 2020 The Authors

Figure EV5. DCX isoforms in human and conservation of key DCX residues in different species.

A Three human splicing variants of DCX are aligned: 360 aa isoform 2 (this study; top sequence), 365 aa isoform 1 (middle sequence), and the 366 isoform (bottom). Sequence differences are boxed and NDC and CDC domain boundaries are indicated with the blue and yellow background, respectively.

B Schematic drawing of the major parts of the brain in different vertebrate classes, with an arrow indicating the relative forebrain growth.

C, D Alignments of NDC and CDC sequences, respectively, in selected vertebrate species. Residues identified as critically important for DCX function through the analysis human mutations data (Appendix Table S1) have been highlighted with red and divergent residues are marked with levels of gray, with most divergent sites colored black. The degree of conservation is expressed with blue and yellow background intensity for NDC (in C) and CDC (in D), respectively: the fainter the color the weaker the conservation. The most divergent site is residue 110 in NDC's loop 4. It is one of the furthest residues from MT lattice in NDC, with a side chain projecting outward. Species selection includes model animals and an example outlier (koala) identified through large alignments of DCX sequences (>114 orthologs found in 183 species, including invertebrates) available through Ensembl genome database (ensembl.org). Koala was is one of the most divergent mammals regarding the DC domain sequences. Three fish species are shown to confirm fish class divergence (the only vertebrate class with dominant midbrain, as shown in A) from other vertebrate classes. Human, *Homo sapiens*; chimpanzee, *Pan troglodytes*; pig, *Sus scrofa*; mouse, *Mus musculus*; koala, *Phascolarctos cinereus*; chicken, *Gallus gallus*; turtle (painted turtle), *Chrysemys picta bellii*; frog, *Xenopus tropicalis*; fugu, *Takifugu rubripes*; tetraodon, *Tetraodon nigroviridis*; tilapia, *Oreochromis niloticus*.

© 2020 The Authors

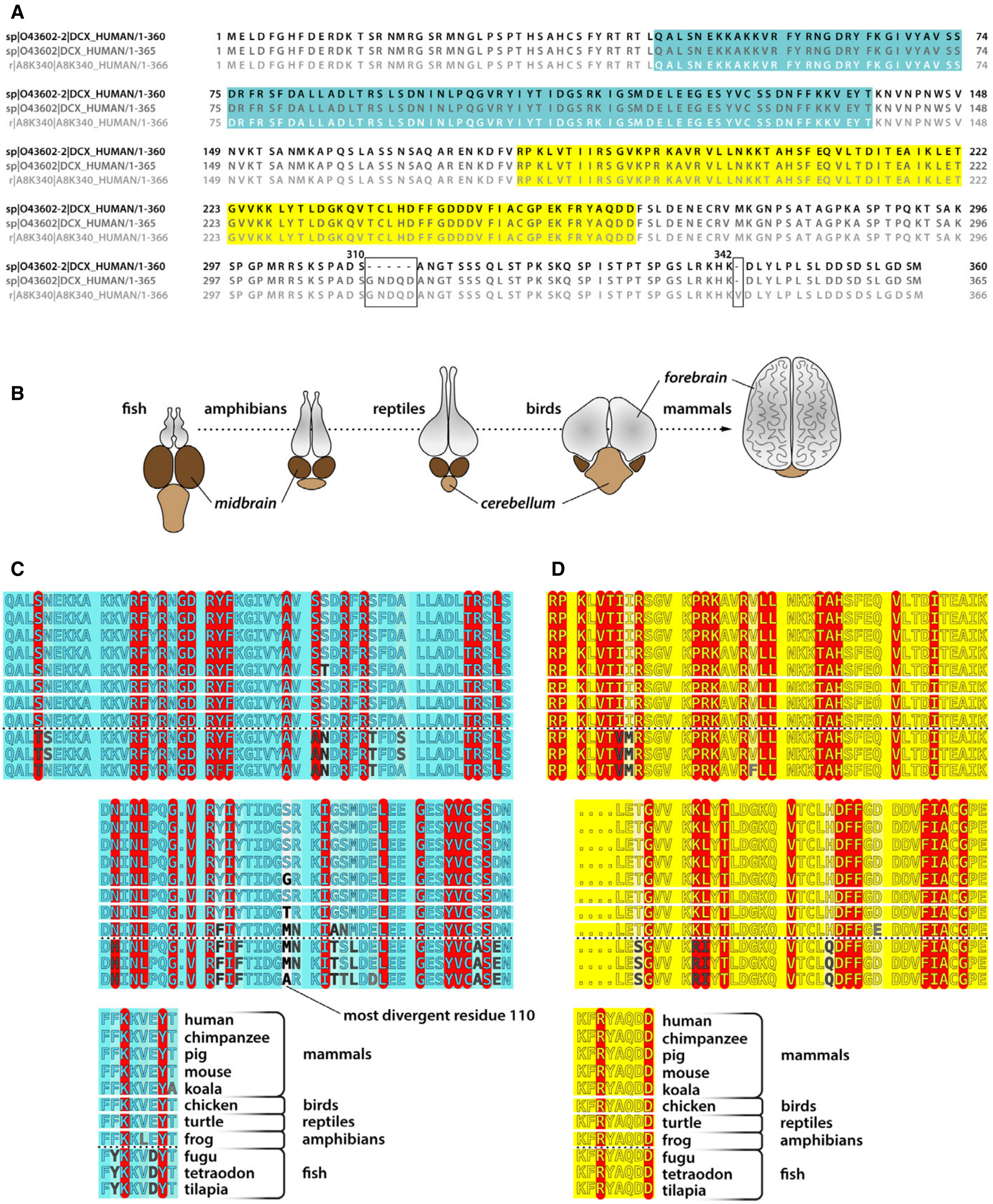

Figure EV5.

 © 2020 The Authors
